# The *Piriformospora indica* effector PIIN_08944 promotes the mutualistic Sebacinalean symbiosis

**DOI:** 10.3389/fpls.2015.00906

**Published:** 2015-10-26

**Authors:** Fidele N. Akum, Jens Steinbrenner, Dagmar Biedenkopf, Jafargholi Imani, Karl-Heinz Kogel

**Affiliations:** Institute of Phytopathology, Research Center for BioSystems, Land Use and Nutrition, Justus Liebig University GiessenGiessen, Germany

**Keywords:** small secreted proteins, root, fungal effectors, endophyte, mutualist, symbiosis, *Piriformospora indica*

## Abstract

Pathogenic and mutualistic microbes actively suppress plant defense by secreting effector proteins to manipulate the host responses for their own benefit. Current knowledge about fungal effectors has been mainly derived from biotrophic and hemibiotrophic plant pathogenic fungi and oomycetes with restricted host range. We studied colonization strategies of the root endophytic basidiomycete *Piriformospora indica* that colonizes a wide range of plant species thereby establishing long-term mutualistic relationships. The release of *P. indica*’s genome helped to identify hundreds of genes coding for candidate effectors and provides an opportunity to investigate the role of those proteins in a mutualistic symbiosis. We demonstrate that the candidate effector PIIN_08944 plays a crucial role during fungal colonization of *Arabidopsis thaliana* roots. *PIIN_08944* expression was detected during chlamydospore germination, and fungal deletion mutants (Pi_Δ08944_) showed delayed root colonization. Constitutive over-expression of *PIIN_08944* in *Arabidopsis* rescued the delayed colonization phenotype of the deletion mutant. *PIIN_08944*-expressing *Arabidopsis* showed a reduced expression of flg22-induced marker genes of pattern-triggered immunity (PTI) and the salicylic acid (SA) defense pathway, and expression of *PIIN_08944* in barley reduced the burst of reactive oxygen species (ROS) triggered by flg22 and chitin. These data suggest that PIIN_08944 contributes to root colonization by *P. indica* by interfering with SA-mediated basal immune responses of the host plant. Consistent with this, *PIIN_08944*-expressing *Arabidopsis* also supported the growth of the biotrophic oomycete *Hyaloperonospora arabidopsidis* while growth of the necrotrophic fungi *Botrytis cinerea* on *Arabidopsis* and *Fusarium graminearum* on barley was not affected.

## Introduction

During their lifecycle, plant roots are constantly exposed to a multitude of microorganisms in the rhizosphere. These interactions which can either be parasitic or mutualistic have a great impact on agriculture and forest life (Harrison, 1999; Sanders, 2011). Mycorrhizal symbioses, an association between plant roots and soil fungi, and the symbiosis of plants with rhizobia have been the main focus of studies on beneficial plant–microbe interactions. Whereas mycorrhizal symbioses are very abundant in nature and are formed by more than 90% of plant species with diverse soil fungi, rhizobial interactions have a narrow host range. Nevertheless, both interactions are beneficial to their hosts, resulting in an increase in phosphorus and/or nitrogen uptake (Parniske, 2008; Sanders, 2011).

*Piriformospora indica*, a basidiomycete fungus with an endophytic life style, colonizes the roots of a wide variety of monocotyledonous and dicotyledonous plants, including the model plants *Arabidopsis* and barley and establishes a Sebacinalean symbiosis (Varma et al., 1999; Peškan-Berghöfer et al., 2004; Lee et al., 2011; Qiang et al., 2012a; Glaeser et al., 2015). *P. indica* was initially discovered in the Indian Thar desert in northwest Rajasthan and has been extensively investigated for its beneficial effects during interaction with plants. Previous reports have shown that infestation by *P. indica* spores and/or culture filtrates leads to growth promotion, enhanced resistance to biotic and abiotic stresses, increase grain yield, and enhanced phosphate and nitrate uptake (Peškan-Berghöfer et al., 2004; Sherameti et al., 2005; Waller et al., 2005; Deshmukh and Kogel, 2007; Achatz et al., 2010; Yadav et al., 2010). The successful interaction between *P. indica* with its hosts is achieved through an active suppression of part of the host defense responses (Jacobs et al., 2011). The fungus has evolved a dual colonization strategy whereby it initially colonizes living root cortex cells biotrophically (3 dpi) and later switches to a cell death-associated colonization stage (7 and 14 dpi), where it grows mostly on dead plant material (Jacobs et al., 2011; Qiang et al., 2012b; Lahrmann et al., 2013). As a consequence, the level of fungal colonization was influenced by the cell death inhibitor protein BAX INHIBITOR 1, at least during the first 3 weeks after colonization of barley roots (Deshmukh et al., 2006). Furthermore, several phytohormones affect plant colonization by *P. indica.* For example, ethylene signaling supports the colonization of barley and *Arabidopsis* roots by *P. indica*. In addition, genes involved in the biosynthesis and signaling of ethylene, auxin, abscisic acid (ABA) and gibberellic acid (GA) were found to be differentially regulated during colonization of barley roots by *P. indica* (Schäfer et al., 2009; Khatabi et al., 2012). While, it has been reported that *P. indica* is indeed capable of producing the auxin indole acetic acid (IAA) in culture medium and also regulate auxin-induced genes in roots, this auxin was not required for growth promotion, but rather supported the biotrophic colonization of barley (Vadassery et al., 2008; Lee et al., 2011; Hilbert et al., 2012).

Similarly to plant pathogenic fungi, mutualistic fungi have also evolved the ability to deliver molecules, called effectors, inside the cells to manipulate the host metabolism, and enhance microbial infection (de Jonge et al., 2010; Kloppholz et al., 2011; Plett et al., 2011; Rafiqi et al., 2012). Effectors, which can exert their actions either in the host cytoplasm or apoplast, often lack conserved domains and are usually under high selective pressure from the host (Dodds et al., 2009; de Jonge et al., 2010; Rafiqi et al., 2010). While effectors from pathogenic fungi have been extensively studied, little is known about effectors secreted by mutualistic symbionts. Two independent studies, reported the involvement of mutualistic fungal effector proteins in the establishment and maintenance of symbiosis in endo- and ectomycorrhiza, allowing the fungus to manipulate the plant defense response in both cases (Kloppholz et al., 2011; Plett et al., 2011, 2014). The release of the *P. indica* genome (Zuccaro et al., 2011) allowed the identification of hundreds of genes coding for small secreted proteins (SSPs < 300 aa). These proteins are considered candidate effectors. Twenty five of *P. indica’s* effector candidates contain a highly conserved pattern of seven amino acid “RSIDELD” at the C-terminus collectively known as the DELD effectors. Although it is speculated that the DELD motif could function in effector translocation in a similar way as the RXLR and LFLAK motifs found in oomycete effectors (Whisson et al., 2007; Schornack et al., 2010) and/or in signaling, the exact function of the DELD motifs is still unknown. While several *P. indica* effector candidates, including some of the DELD effectors, are induced exclusively either in *Arabidopsis* or barley and thus behave host specifically, a small group were expressed in both *Arabidopsis* and barley (i.e., host unspecific; Lahrmann et al., 2013). Despite the fact that several *P. indica* effector candidates including some of the DELD effectors were up-regulated *in planta* (Zuccaro et al., 2011) their virulence functions during the interaction between *P. indica* with plants are largely unknown, opening new avenues to investigate their role in the Sebacinalean symbiosis.

In this study, we investigate the function of the *P. indica* effector candidate PIIN_08944, a non DELD effector, during the interaction of plants with *P. indica*. We show that the candidate effector contributes to plant colonization by the mutualistic fungus by suppressing the salicylate (SA)-mediated basal resistance response. Moreover, we found that PIIN_08944 also supports growth of the biotrophic oomycete *Hyaloperonospora arabidopsidis*, while growth of the necrotrophic fungi *Botrytis cinerea* and *Fusarium graminearum* were unaffected.

## Materials and Methods

### Fungal and Plant Materials and Root Inoculation

*Piriformospora indica* (Verma et al., 1998) cultures (DSM11827, Deutsche Sammlung für Mikroorganismen und Zellkulturen, DSMZ, Braunschweig, Germany) were propagated at 28°C in liquid complete medium (CM; Pham et al., 2004) supplemented with 2% glucose on a shaker at 140 rpm. For solid medium, 1.5% agar was added to the CM medium. *Hygromycin B* (100 μg/ml) was supplemented for growth of *P. indica* transformants.

Barley seeds *(Hordeum vulgare* cv. Golden Promise) were surface sterilized with 70% ethanol for 2 min, 12% sodium hypochlorite for 1.5 h, and washed with sterile distilled water for 3 h. Sterilized seeds were kept in the dark for 3 days on sterile wet filter paper at room temperature. For colonization studies, 3-day-old barley seedlings were transferred into sterile jars containing 1/10 PNM medium (Basiewicz et al., 2012) supplemented with 0.4% (w/v) GELRITE (Duchefa) and inoculated with *P. indica* chlamydospore suspension (500,000 chlamydospore mL^-1^ in 0.002% TWEEN20). Inoculated plants were transferred to a growth chamber and grown under a dark/light cycle of 16 h light (110 μmol m^-2^ s^-^1) at 24°C and 8 h dark at 18°C. Control plants were treated with water containing 0.002% (v/v) TWEEN20. Root samples were collected at the indicated time points and immediately frozen in liquid nitrogen.

*Arabidopsis thaliana* seeds (ecotype Columbia-0, col-0) were surface sterilized with 70% ethanol for 1 min, for 10 min with 6% sodium hypochlorite, and washed six times for 5 min in sterile water. Seeds were placed on *A. thaliana* medium + sucrose (ATS) medium (5 mM KNO_3_, 2.5 mM KH_2_PO_4_ 2 mM MgSO_4_, 2 mM Ca(NO_3_)_2_, 50 μM Fe-EDTA 70 μM H _3_BO_3_, 14 μM MnCI_2_, 0.5 μM CuSO_4_, 1 μM ZnSO_4_, 0.2 μM NaMoO_4_, 10 μM NaCl, 0.01 μM CoCl_2_; Estelle and Somerville, 1987) containing 0.4% (w/v) GELRITE (Duchefa) and grown vertically in square petri dishes under a dark/light cycle of 16 h light (110 μmol m^-2^ s^-1^) at 23°C and 8 h dark at 18°C. For inoculation, *Arabidopsis* roots of 7-day-old seedlings were inoculated with 1 ml of 500,000 chlamydospores mL^-1^. Root material was harvested and immediately frozen in liquid nitrogen at 3, 7, 14, and 21 days post inoculation (dpi). For each time point, roots from 80 to 100 plants were harvested. For all root inoculation experiments, chlamydospores were collected from 3 to 4-week-old *P. indica* cultures grown on solid CM medium using water containing 0.002% (v/v) TWEEN20.

### Plasmid Construction and Generation of Transgenic *Arabidopsis* and Barley Plants

*PIIN_08944* lacking the predicted signal peptide was amplified from cDNA by PCR using primers GS-08944dSP-F and SmaI-08944-R (**Supplementary Table S1**). For cDNA synthesis, total RNA from *P. indica* was extracted using TRIzol (Invitrogen); cDNA was synthesized using the qScript cDNA synthesis kit (Quanta Biosciences) following the manufacturer’s instructions. For the generation of *GFP-PIIN_08944* fusion construct, GFP (Green Fluorescence Protein) was amplified using primers SmaI-GFP-F and ATG-GSGFP-R (**Supplementary Table S1**) and the PCR products of *PIIN_08944* and *GFP* were fused by overlap extension PCR. The fusion construct was ligated at the SmaI site in the pUBI-AB vector^1^ (DNA Cloning Service, Hamburg, Germany) and the entire cassette was inserted at the SfII restriction site into the binary vector pLH6000^2^ (DNA Cloning Service, Hamburg, Germany) under the control of the maize ubiquitin promoter or the SmaI restriction site in the expression vector pICH (Weber et al., 2011) under the control of the constitutive CaMV35S promoter. The resulting plasmids pLH6000 UBI::GFP-8944 and pICH CaMV35S::8944 were confirmed by sequencing (Sequence Laboratories Göttingen, Germany) before plant transformation. For *Arabidopsis* transformation, the plasmid pICH CaMV35S::8944 was introduced into *Agrobacterium tumefaciens* strain GV3101 by electroporation and selected on LB medium containing kanamycin (25 μg/ml), gentamycin (20 μg/ml), and rifampicin (15 μg/ml) antibiotics. Transformation and regeneration of *Arabidopsis* was performed by floral dip (Clough and Bent, 1998) and transformants were selected on MS (Murashige and Skoog) medium containing kanamycin (25 μg/ml). For barley transformation, the pLH6000 UBI::GFP-8944 plasmid was used. Transgenic barley plants were generated as described (Imani et al., 2011).

### DNA, RNA Extraction and Quantitative Real-time PCR

For genomic DNA and total RNA extraction, plant and fungal materials were powdered under liquid nitrogen using a mortar and pestle. Genomic DNA was isolated following the method of Doyle and Doyle (Doyle, 1987). For gene expression studies, seedlings were grown on solid half strength MS medium for 2 weeks and then transferred in six-well-plates containing liquid half-strength MS medium. After a recovery phase of 3 days, flg22 (Davids Biotechnology, Germany) was pipetted to each well containing MS medium with seedlings in a final concentration of 100 nM flg22 and harvested after 0, 2, 6, and 12 h. For all experiments, total RNA was extracted from plant or fungal materials using TRIzol (Invitrogen), and aliquots were used for cDNA synthesis with the qScript cDNA synthesis kit (Quanta Biosciences). Forty nanograms of genomic DNA or cDNA were used as template for quantitative real-time PCR (qPCR) analysis, using the SYBR Green JumpStart Taq ReadyMix (Sigma–Aldrich) and the 7500 FAST Real-Time PCR System under standard conditions (Applied Biosystems). For the detection of fungal and plant DNA, the primers ITS_F/ITS_R and UBQ4_F/UBQ4_R were used (**Supplementary Table S1**). The relative fungal genomic DNA or relative gene expression were calculated using the 2^-ΔCT^ method (Livak and Schmittgen, 2001).

### Construction of Knockout Vector, *P. indica* Transformation

In order to generate a genomic deletion of the *PIIN_08944* gene, a hygromycin B resistance cassette was introduced by homologous recombination at the *PIIN_08944* locus. A 180 bp fragment (5′ UTR region), upstream and a 1000 bp fragment (3′ UTR region), downstream of *PIIN_08944* was amplified by PCR using the primers KpnI_US_8944F/KpnI_US_8944R and StuI_DS_8944F/SacI_DS_8944R, respectively. The fragments were cloned into the flanking KpnI and StuI/SacI restriction sites of the hygromycin B resistance cassette into the pHSP70 vector derived from the pbshhn-Tef vector (Kamper, 2004) by replacing the TEF promoter with the Hsp70 promoter. The constructed plasmid was confirmed by sequencing (Sequence Laboratories Göttingen, Germany) and used to transform *P. indica* following the PEG mediated transformation protocol (Hilbert et al., 2012).

### Southern Blot

To verify integration of the hygromycin resistance cassette into the nuclear genome of *P. indica*, Southern blot analysis was performed. Genomic DNA from 7-day-old cultures grown on CM medium was extracted; 10–20 μg of extracted DNA was digested overnight with *Sac*I (NEB). The digested DNA was separated on 0.9% TAE agarose gel for 5 h at 80 V and blotted onto a nylon membrane (AmershamBiosciences Hybond-N+, GE Healthcare) over night. The DNA was UV cross-linked to the membrane in a GS GENE LINKER UV chamber (BIO-RAD) using an auto cross-linking program (C2, 50 mμ Joule). The labeling of the Hygromycin B probe was performed using the Prime-a-Gene^®^ Labeling System according to the manufacturer’s instructions (Promega). Hybridization and washing steps were performed at 65°C. The membrane was exposed on phosphorimaging screens (Bio-Rad) and signals were detected using a molecular imager and the Quantity One software (Bio-Rad).

### PIIN_8944 Transcript Analysis

To assess the disruption of the *PIIN_08944* gene in *P. indica*, mRNA expression analysis was performed using reverse transcriptase-PCR (RT-PCR). RNA was extracted from 7-day-old *P. indica* cultures grown on CM medium/plates or from *Arabidopsis* roots inoculated with the *P. indica* deletion mutant, harvested at 3, 7, 14, and 21 dpi. cDNA was synthesized from total RNA and 40 ng served as template for RT-PCR in a final volume of 25 μl, and a thermal cycling period of 30 cycles. The *P. indica* ubiquitin gene *PIIN_01523* served as a reference.

### Plant Infection Assays

For *Arabidopsis* leaf inoculation, *B. cinerea* strain B05.10 (Rui and Hahn, 2007) was cultured on HA agar as described (Doehlemann et al., 2006); conidia concentration was adjusted to 2 × 10^5^ conidia per mL^-1^ in potato dextrose broth (PDB). Fifteen rosette leaves were detached from 10 different 4-week-old plants and transferred into square petri plates containing 1% agar. Five μl droplets of the conidial suspension was pipetted onto each side of the middle vein and incubated during a 12 h photoperiod at 22°C. Disease symptom progression was analyzed by measuring the lesion size (in centimeter) at 5 dpi from the digital images using the Image J free software program^3^ by calculating the percentage of leaf area showing disease symptoms relative to the non-inoculated area. Two week old *Arabidopsis* seedlings were sprayed with the *H. arabidopsidis* isolate Noks1 (Holub and Beynon, 1997) at a spore concentration of 30,000 spores mL^-1^. Infection development was scored 4 days after infection by counting sporangiophores on true leaves (Tome et al., 2014).

For barley leaf inoculation, *F. graminearum*, Fg-IFA65 (Jansen et al., 2005) was cultured on SNA agar as described (Koch et al., 2013) and conidia concentration was adjusted to 5 × 10^5^ mL^-1^ in 0.02% Tween 20. Leaves of 3-week-old barley of each transgenic line (T2 generation) and empty vector (EV) control plants were detached and transferred into square petri plates containing 1% agar. Each leaf was inoculated with 20 μl and plates were incubated at 22°C with a 16 h photoperiod for up to 6 days. Disease symptoms were analyzed by measuring the lesion size/area using the Image J free software program^3^ and the relative amount of fungal biomass determined by qPCR after DNA extraction using the β-tubulin gene (FGSG_09530) as normalization control.

For barley root infection, roots of 3-day-old seedlings were dip-inoculated in a *F. graminearum* conidial suspension for 30 min, then transferred into pots containing a 2:1 mixture of expanded clay (Seramis, Masterfoods, Verden, Germany) and Oil-Dri (Damolin, Mettmann, Germany) and allowed to grow in a growth chamber under long day conditions (dark/light cycle of 16 h light (110 μmol m^-2^ s^-^1) at 24°C and 8 h dark at 18°C) for up to 2 weeks. Plants were carefully collected, roots washed with H_2_O and the root length (in cm) and root fresh weight (in mg) were determined.

### Measurement of ROS

Leaf disks from 3-week-old barley plants (HvPIIN_08944 or EV control) or 4-week-old *Arabidopsis* plants (AtPIIN_08944 or WT control) were cut and pre-incubated overnight in sterile distil water in 96 well micro-titer plate. Water is carefully discarded and the leaf disks are treated with luminol (Sigma, A8511-5 g) 40 μl luminol buffer [15 mg/ml], 400 μl horseradish peroxidase [1 mg/ml] and elicitor [100 nM flg22, 200 mg/ml crab shell chitin (sigma–aldrich) or water control]. Immediately after treatment, luminescence was measured in a TECAN infinite^®^ F200 micro plate reader (TECAN, Switzerland). The relative light units over time as a result of the production of oxygen radicals were measured for 50 min.

### Statistical Analysis

All experiments were repeated at least two to three times in each case as indicated in the figure legends. A student’s two-tailed t test was used to determine the significance of the data reported in this study. Differences were considered to be significant at *P* ≤ 0.05.

## Results

### *In silico* Analysis of the *P. indica* Effector Candidate PIIN_08944

The genome of *P. indica* revealed the existence of 543 small secreted proteins (Zuccaro et al., 2011; Rafiqi et al., 2013). PIIN_08944 is a small secreted protein containing 120 amino acids (aa; GenBank:CCA74964.1) that lacks cysteine residues. The presence of an N-terminal signal peptide has been a main criterion for selecting fungal genes coding for putative effectors. Using SignalP 4.0 (Petersen et al., 2011), PIIN_08944 was predicted to contain a 23 aa signal peptide located at the N-terminal (aa 1–23); the TMHMM software (Krogh et al., 2001) failed to detect a transmembrane domain. Search for other conserved domains using the conserved domain database at NCBI^4^ and pfam version 27.0 (EMBL-EBI^5^) did not reveal any conserved domains. Moreover the protein did not show a significant sequence similarity to proteins with known functions of other organisms.

### *PIIN_08944* is Expressed During Colonization of *Arabidopsis* Roots

**FIGURE 1:**
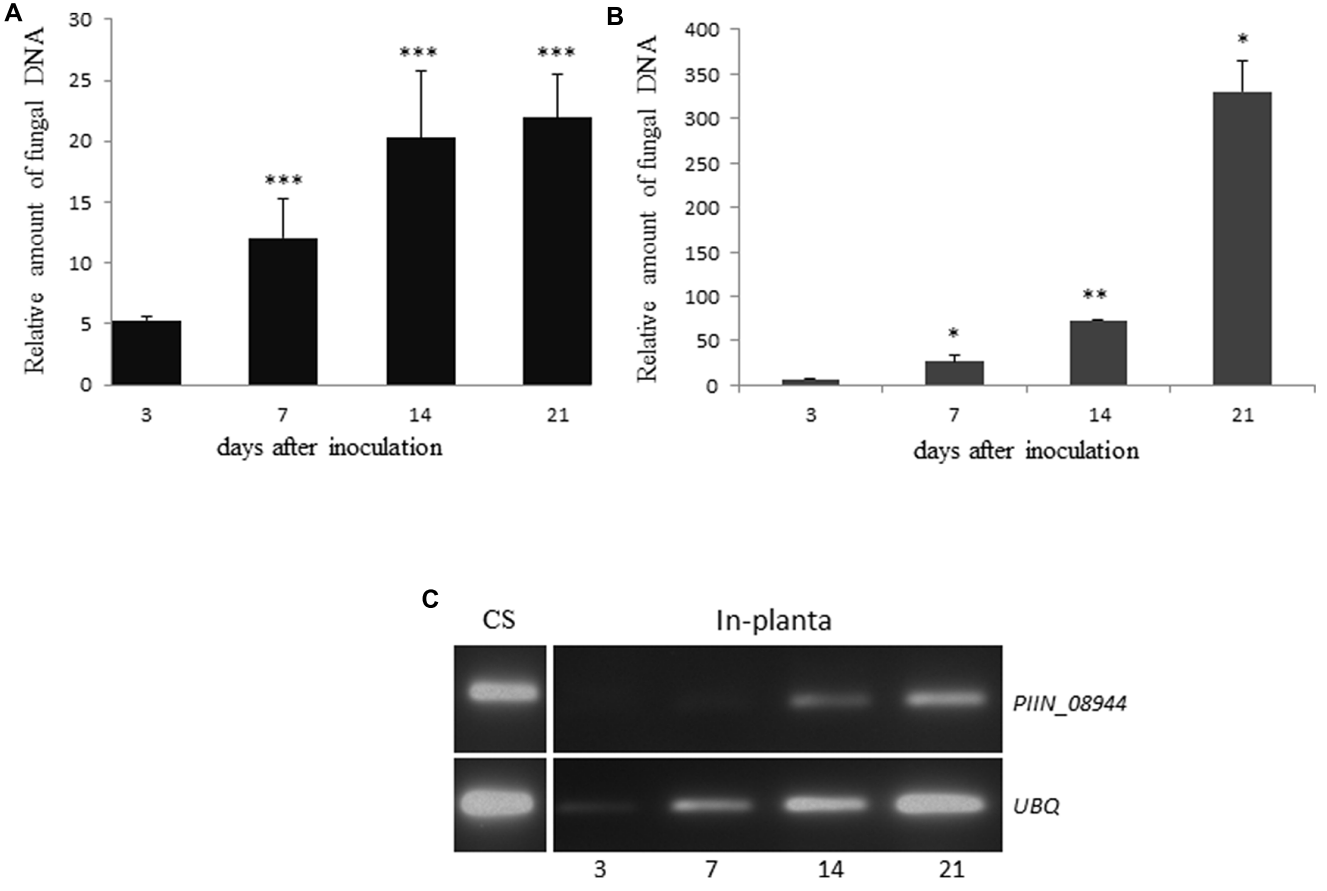
**Colonization of plant roots by *Piriformospora indica* increases over time.** **(A)** Roots of 3-day-old barley grown on 1/10 PNM agar in sterile glass jars, were inoculated with chlamydospores of *P. indica*. Colonization levels were determined at 3, 7, 14, and 21 dpi as the relative amount of fungal DNA by qPCR using barley (HvUBQ-60-Deg) and fungal (ITS) specific primers. Values represent the mean ± SE of two independent experiments. **(B)** Seven-day-old *Arabidopsis* seedlings were inoculated with chlamydospores of *P. indica*. Colonization levels were determined at 3, 7, 14, and 21 dpi as the relative amount of fungal DNA by qPCR using *Arabidopsis* (*At*UBQ4) and fungal (ITS) specific primers. Data represents the Ct thresholds of ITS relative to the Ct thresholds of *At*UBQ-4 (±SE obtain from three technical replicates of one biological experiment). Experiments were repeated twice with similar results. Asterisks indicate significance between time points at ^∗^*P* < 0.05, ^∗∗^*P* < 0.01, ^∗∗∗^*P* < 0.001 analyzed by student’s *t-*test. **(C)** Analysis of *PIIN_08944* expression by semi-quantitative RT-PCR. Transcripts of *PIIN_08944* were detected in *in vitro* germinated *P. indica* chlamydospores (CS) grown in CM liquid medium for 7 days and *in planta* during colonization of *Arabidopsis* roots by *P. indica* by RT-PCR. Transcript abundance increased over time from 3 to 21 dpi. The *P. indica ubiquitin (UBQ)* gene served as reference.

To confirm previous results and to benchmark our colonization assay, we investigated the increase in fungal biomass during *P. indica* colonization of *Arabidopsis* and barley roots by using qPCR. Similar colonization pattern were observed for both plant species over a time period of 21 days. The relative amount of fungal DNA increased in roots from 3 to 21 dpi, with a more than 20-fold increase in barley (**Figure 1A**) and 300-fold increase in *Arabidopsis* (**Figure 1B**) relative to plant DNA. This is in agreement with published results (Pedrotti et al., 2013).

Based on microarray expression datasets, several of *P. indica’s* effector candidates, including the DELDs are strongly expressed during symbiosis (Zuccaro et al., 2011). To determine the expression pattern of *PIIN_08944*, we reviewed the literature and a public available dataset from ArrayExpress^6^(E-GEOD-47775) for *PIIN_08944* and compared it to *P. indica*’s effector candidates that contain the DELD motif (DELD effectors; Zuccaro et al., 2011). *PIIN_08944* showed very high levels of expression (log_2_ of 11.4) compared to *DELD* genes and therefore ranked on the fourth position after candidate effectors *PIIN_09226*, *PIIN_00561*, *PIIN_05098*, and *PIIN_09643*. *PIIN_08944* is found in the top 20% of all expressed genes of *P. indica* grown under minimal PNM medium (Lahrmann et al., 2013). Since the published time-course experiment did not reveal any significant differential expression at 3 and 14 dpi (Zuccaro et al., 2011; Lahrmann et al., 2013, Lahrmann, 2014), we expanded our analysis to 21 dpi and examined the expression profile of *PIIN_08944* by RT-PCR (**Figure 1C**). *PIIN_08944* transcripts were detected in RNA from *in vitro* germinated *P. indica* chlamydospores as well as colonized *Arabidopsis* roots. The increase in transcript abundance from 3 to 21 days is correlated with the increase in fungal biomass as the ubiquitin reference gene *(PIIN_01523)* also increased in abundance over the time course. This result is in agreement with our analysis of the public available microarray data where *PIIN_08944* did not show differential regulation.

### *P. indica* Deletion Mutants (Pi_Δ08944_) Show Reduced Colonization of *Arabidopsis* Roots

**FIGURE 2:**
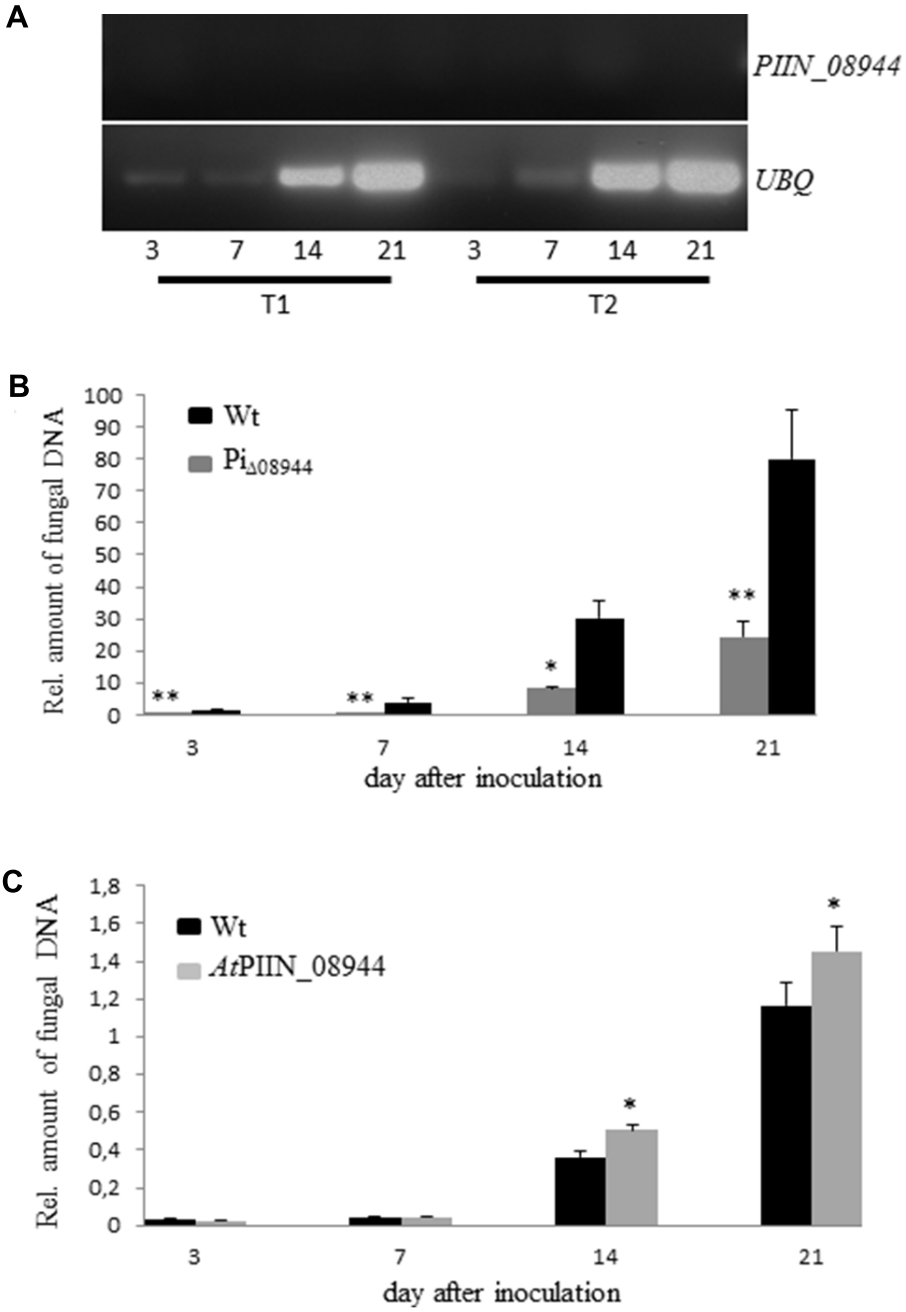
**Characterization of *P. indica* Knockout Strains Pi_Δ08944_ (*Pi*T1-*Pi*T5).** **(A)** Roots of 7-day-old *Arabidopsis* seedlings were inoculated with chlamydospores of Pi_Δ08944_ strains *Pi*T1 or *Pi*T2, respectively. The roots were harvested at 3, 7, 14, and 21 dpi and *PIIN_08944* transcript levels were determined from extracted RNA by RT-PCR using *PIIN_08944* specific primer. *PIIN_08944* transcripts from Pi_Δ08944_ strains were not detected *in planta*. The *P. indica ubiquitin UBQ* gene served as reference. **(B)** Deletion of *PIIN_08944* delays colonization of *Arabidopsis* roots. Roots of 7-day-old *Arabidopsis* seedlings were inoculated with Pi_Δ08944_ (*Pi*T1) and wt, respectively. **(C)** Colonization of *PIIN_08944*-expressing *Arabidopsis* with Pi_Δ08944_ (*Pi*T1) mutant. Roots of 7-day-old AtPIIN_08944OE seedlings or wt were inoculated with Pi_Δ08944_. Fungal biomass was determined at 3, 7, 14, and 21 dpi as relative amount of fungal DNA by qPCR using fungal (ITS) and plant (*At*UBQ4) specific primers. Data displays the Ct thresholds of ITS relative to the Ct thresholds of *At*UBQ- 4 (±SE obtain from three technical replicates of one biological experiment). Experiments were repeated twice with similar results. Asterisks indicate significance at ^∗^*P* < 0.05, ^∗∗^*P* < 0.01 analyzed by Student’s *t*-test.

To investigate the impact of PIIN_08944 on root colonization, deletion mutants of *P. indica* lacking *PIIN_08944* (Pi_Δ08944_) were generated using homologous gene replacement. After transformation of *P. indica* protoplasts and selection on hygromycin containing CM medium, five transformants were recovered. RT-PCR on RNA extracted from cultures grown on CM medium confirmed deletion of *PIIN_08944* as evident by the absence of transcripts (**Supplementary Figure S4A**). To further confirm that *PIIN_08944* expression was not induced in the mutants during root colonization, *Arabidopsis* roots were inoculated with all five *P. indica* deletion mutants and RT-PCR analysis was performed after roots were harvested at 3, 7, 14, and 21 dpi. In two Pi_Δ08944_ mutants (strains *Pi*T1 and *Pi*T2), transcripts could not be detected at all analyzed time points (**Figure 2A**). To elucidate the number of insertion of the hygromycin resistance cassette in the genome of *P. indica* we performed southern blot analysis. *Pi*T1 contained two copies and *Pi*T2 contained one copy of T-DNA (**Supplementary Figure S4B**).

To assess whether the deletion of *PIIN_08944* has any effect on the growth rate of *P. indica*, we monitored the progression of growth of *Pi*T1 and *Pi*T2 on CM plates over a period of 21 days and compared it to the wild type (wt). The deletion of *PIIN_08944* did not affect the colony morphology (**Supplementary Figure S1**) and the growth rate of *P. indica* (data not shown). However, when *Arabidopsis* roots were inoculated with Pi_Δ08944_ (*Pi*T1), colonization was significantly delayed compared to roots inoculated with the wt strain over the analyzed period of 21 days (**Figure 2B**), demonstrating that PIIN_08944 likely plays a crucial role in the colonization process of roots by *P. indica.*

### *In Planta* Expression of PIIN_08944 Rescues the Delayed Growth Phenotype of Pi_Δ08944_

To investigate whether PIIN_08944 is able to rescue the delayed colonization phenotype of Pi_Δ08944,_ we generated *Arabidopsis* plants expressing *PIIN_08944* under the control of the constitutive 35 s CaMV promoter (AtPIIN_08944OE plants). The integration of the *PIIN_08944* transgene was confirmed by PCR analysis. Several independent AtPIIN_08944OE (T1 generation) plants were selected and self-pollinated to produce T2 generation lines. RT-PCR analysis confirmed that *PIIN_08944* was highly expressed in independent T2 transgenic plants (**Supplementary Figure S4C**). No difference in plant growth was observed between AtPIIN_08944OE vs. the corresponding wt plants (data not shown). To further expand this finding, transgenic barley plants expressing a fusion between *PIIN_08944* and *GFP* under the constitutive maize ubiquitin promoter were generated (HvPIIN_08944OE plants). Genomic insertion of a construct coding for a GFP:PIIN_08944 fusion protein was confirmed by PCR analysis and accumulation of GFP:PIIN_08944 in transgenic barley lines (T2 generation) was confirmed using western blot analysis. The analysis showed that GFP:PIIN_08944 was correctly expressed and no truncated versions were produced except for line 19 (**Supplementary Figure S2**). Consistent with the above finding, no difference in plant growth was observed between HvPIIN_08944OE vs. the corresponding wt (data not shown). Together these data show that the expression of *PIIN_08944* does not affect development of barley and *Arabidopsis*. When roots of AtPIIN_08944OE plants were inoculated with Pi_Δ08944_, colonization rate was similar in AtPIIN_08944OE vs. wt plants at 3 and 7 dpi. At 14 and 21 dpi colonization was higher in AtPIIN_08944OE plants vs. wt (**Figure 2C**). Additionally, HvPIIN_08944OE plants showed enhanced root colonization compared to wt barley, when inoculated by *P. indica* (**Supplementary Figure S3**). These data suggest that PIIN_08944 supports *P. indica*’s colonization during interaction with plants.

### PIIN_08944 Interferes with Basal Defense Response in *Arabidopsis* and Barley

**FIGURE 3:**
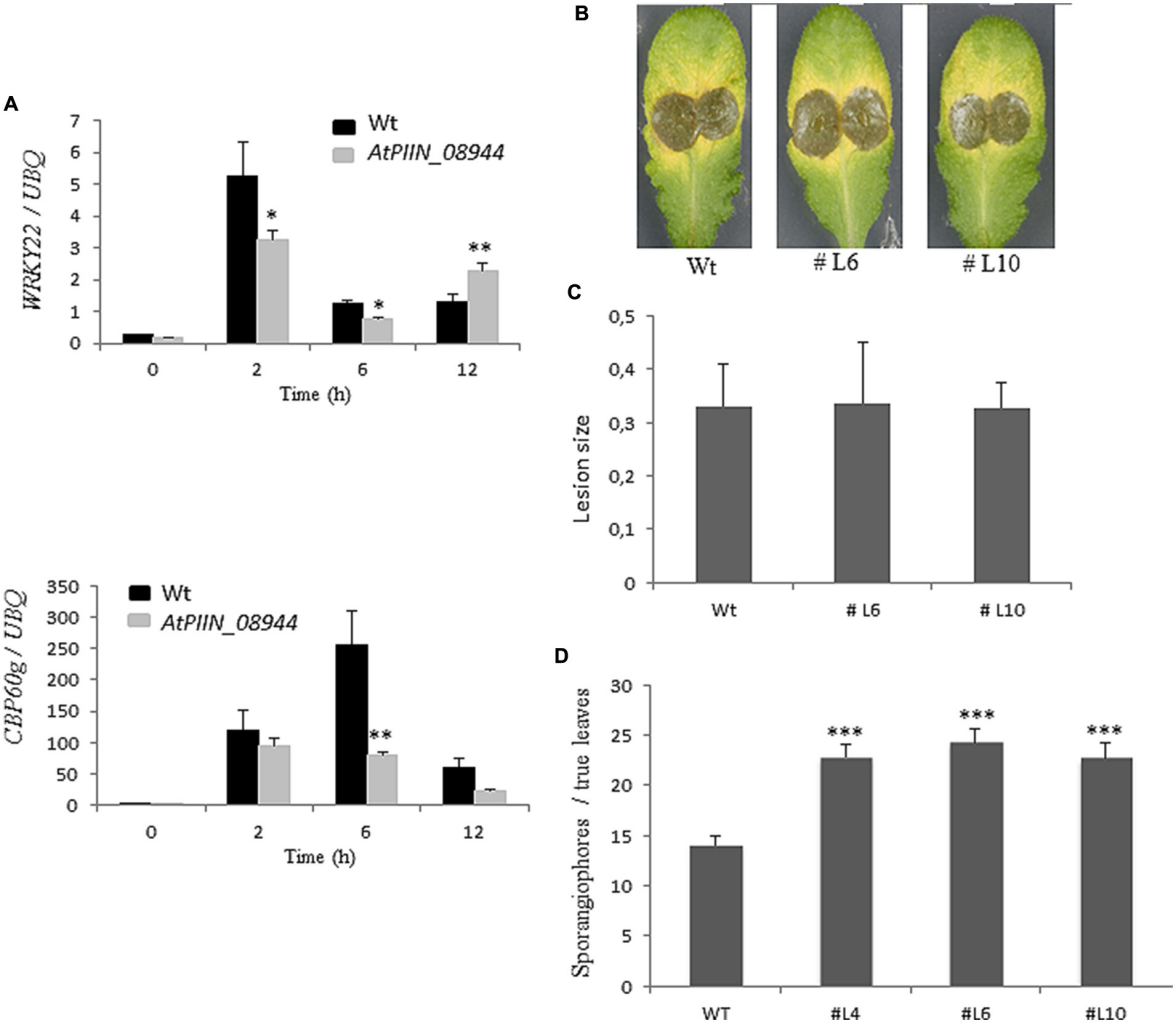
**Assessment of the immune status of AtPIIN_08944OE plants.** **(A)** Two-week-old AtPIIN_08944OE and the respective wt plants were treated with 100 nM flg22 in six well plates. RNA was extracted at 0 (untreated control), 2, 6, and 12 hpt and qPCR was performed. Suppression of flg22-induced transcription was observed for *AtWRKY22* and *CBP60g*. *Arabidopsis UBQ-4* was used for normalization. Values are means ± SE of two independent experiments. **(B)** Detached leaves of four to 6-week-old AtPIIN_08944OE (lines #L6, #L10) and the respective wt plants were inoculated with conidia of *Botrytis cinerea*. Photographs were taken 5 dpi. **(C)** Average lesion size of AtPIIN_08944OE (lines #L6 and #L10) and the wt plants after inoculation with *B. cinerea*. Averages were calculated from 10 to 15 leaves per line. Values are means ± SE of three independent experiments. **(D)** Two-week-old AtPIIN_08944OE seedlings (lines #4 #L6 #L10) and wt plants were sprayed with spores of *Hyaloperonospora arabidopsidis* and sporangiophores development on true leaves determined 4 dpi. For each line at least 100 plants were analyzed. Values are means ± SE of two independent experiments. Asterisks indicate significant differences at ^∗^*P* < 0.05, ^∗∗^*P* < 0.01, ^∗∗∗^*P* < 0.001 as analyzed by student’s *t-*test.

Previous reports showed that jasmonic acid (JA) supports colonization of *Arabidopsis* roots by *P. indica* (Jacobs et al., 2011). The same report showed that *P. indica* inhibits plant defense in roots, as evidenced by the strong suppression of the pattern-triggered immunity (PTI) marker *WRKY22* (Colcombet and Hirt, 2008) and the SA marker *CBP60g* (Wang et al., 2009). However, whether *P. indica* deploys effectors to manipulate host hormone signaling has not been reported. Since the *in planta* expression of *PIIN_08944* leads to enhanced colonization by *P. indica*, we speculated that PIIN_08944 might suppress the host immune response to support fungal growth. To address this question, AtPIIN_08944OE and wt seedlings were treated with the peptide flg22, a PAMP (pathogen-associated molecular pattern) derived from bacterial flagellin. After harvesting at 0, 2, 6, and 12 h post treatment (hpt), transcript abundance of *WRKY22* and *CBP60g* were assessed by qPCR. In agreement with previous results (Colcombet and Hirt, 2008; Wang et al., 2009; Jacobs et al., 2011), genes were induced at 2, 6, and/or 12 hpt in the wt plants (control). In contrast, the flg22-induced expression of *CBP60g* and *WRKY22* was suppressed in AtPIIN_08944OE plants (**Figure 3A**).

**FIGURE 4:**
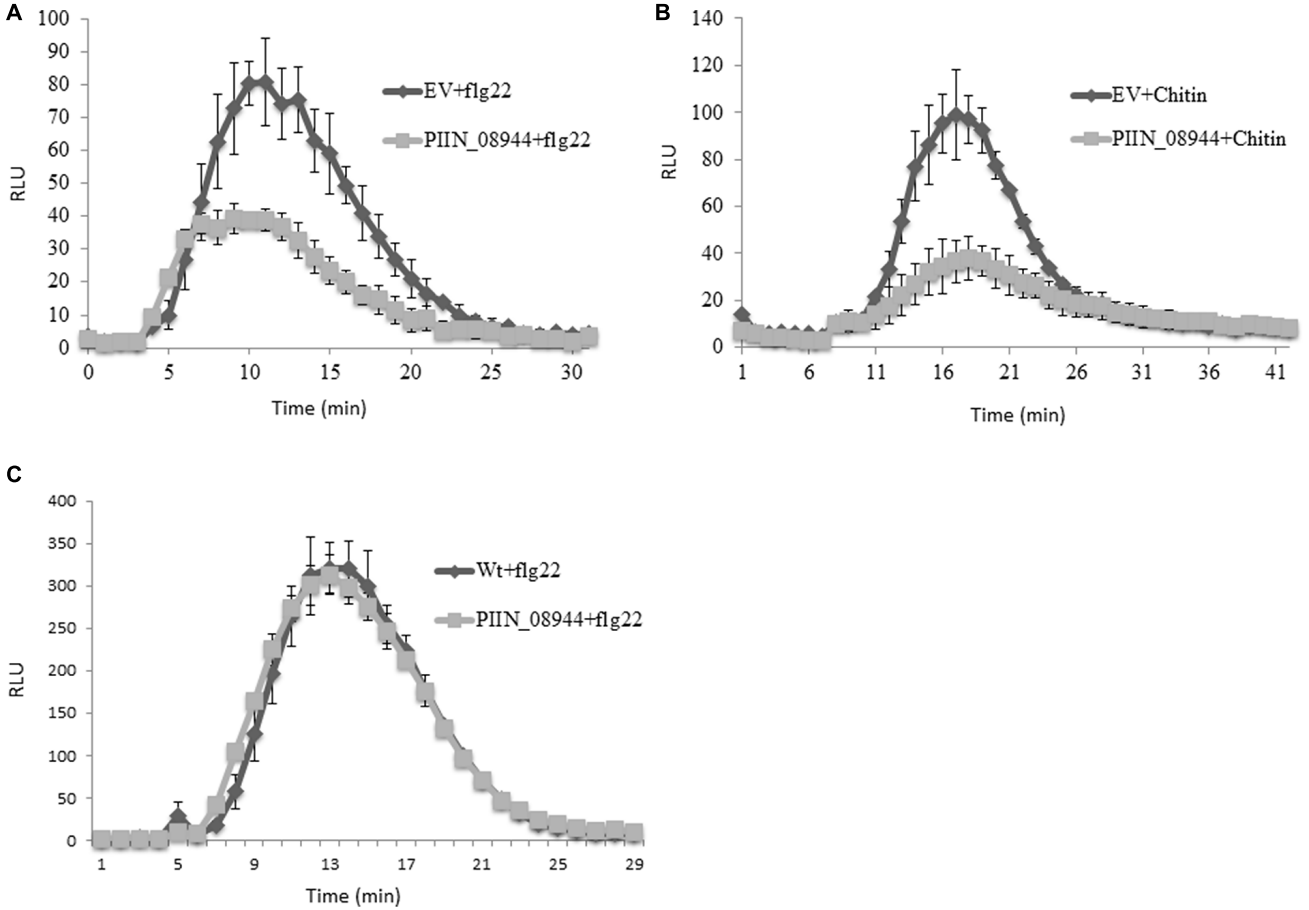
**Assessment of flg22 and chitin-induced reactive oxygen species (ROS) accumulation in barley and *Arabidopsis* tissue.** Leaf disks of 3-week-old barley HvPIIN_08944-OE or EV control plants were treated with **(A)** 100 nM flg22, and **(B)** 200 mg/ml crab shell chitin. **(C)** Leaf disks of 4-week-old AtPIIN_08944-OE or wt *Arabidopsis* plants were treated with 100 nM flg22. ROS was determined by measuring the relative light unit over time with a luminol-chemiluminescence assay using a Tecan reader. Error bars represent the mean ± SE of three independent experiments. (RLU, relative light units; EV, empty vector).

We further analyzed the ability of PIIN_08944 to suppress PTI in barley by analyzing reactive oxygen species (ROS) production after flg22 and chitin treatment. In HvPIIN_08944OE plants, flg22-induced ROS accumulation was reduced to about 50%, while chitin-induced ROS production was almost completely suppressed (**Figures 4A,B**). Notably, however, overexpression of *PIIN_08944* did not suppress flg22-induced ROS production in *Arabidopsis* (**Figure 4C**).

**FIGURE 5:**
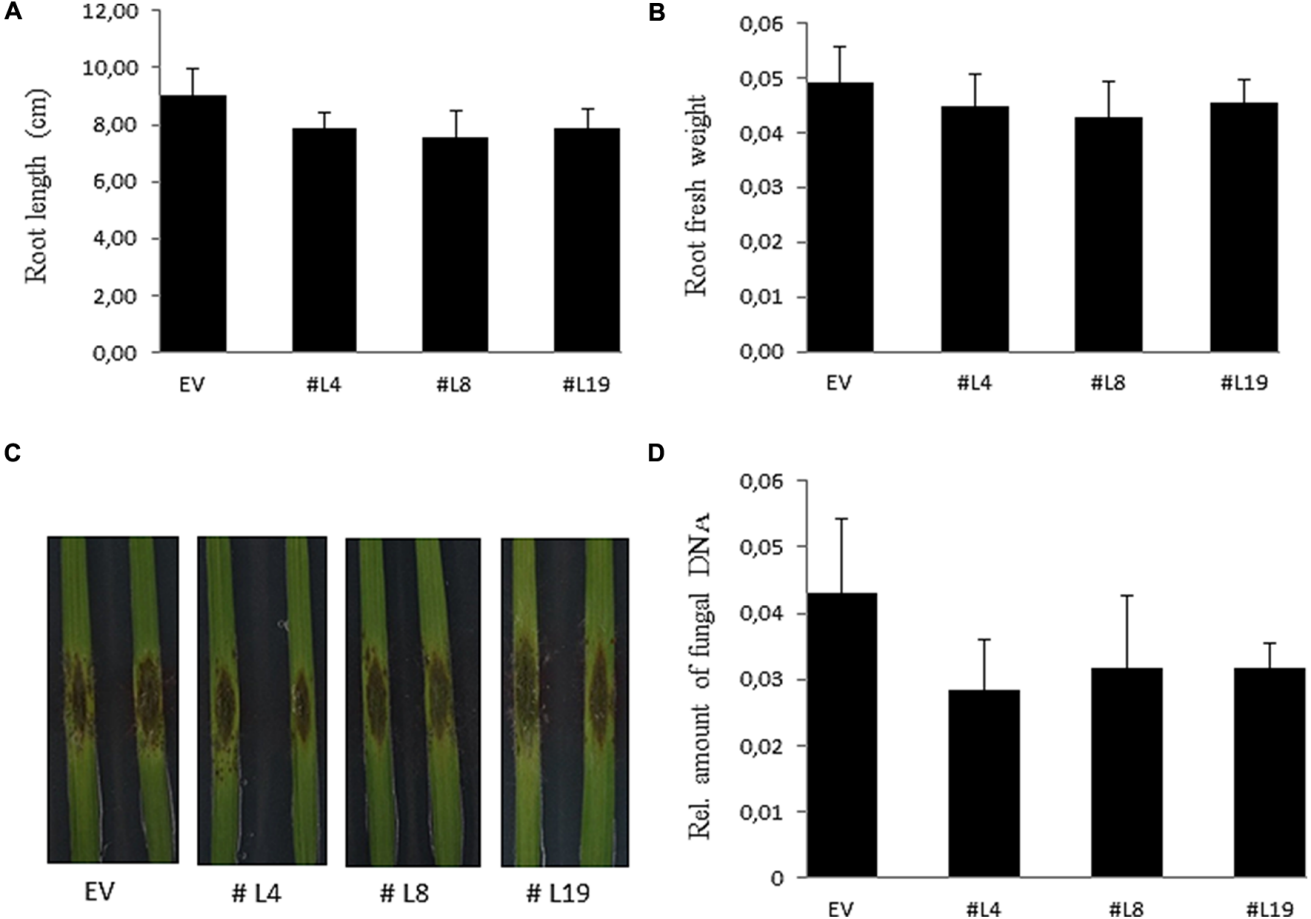
**Characterization of barley HvPIIN_08944OE plants inoculated with macroconidia of *Fusarium graminearum*.** Root length **(A)** and root fresh weight **(B)** of six plants per line inoculated with *F. graminearum*. Values represent the mean with ± SE of three independent experiments. **(C)** Detached leaves of 3-week-old HvPIIN_08944OE plants (#4 #8 #14) and EV control were inoculated with *F. graminearum*. Photographs were taken 6 dpi. The experiment was repeated three times with similar outcome **(D)** Relative fungal biomass on detached HvPIIN_08944OE (#4 #8 #14) and EV control leaves by qPCR. Values represent the mean with ±SE of three independent experiments.

PIIN_08944-enhanced root colonization by *P. indica* and at suppression of PTI responses raised the possibility that plant infection by fungal pathogens could also be affected. To address this question, we tested *PIIN_08944*-overexpressing plants with the necrotrophic ascomycete fungi *B. cinerea (Bc)* and *F. graminearum (Fg)* as well as the obligate biotrophic oomycete pathogen *H. arabidopsidis (Hpa)*. When leaves of 4-week-old transgenic plants were inoculated with *B. cinerea*, no difference in lesion size or infection rate was observed in AtPIIN_08944OE vs. wt plants at 5 dpi (**Figures 3B,C**). Similarly, when HvPIIN_08944OE plants were inoculated with *F. graminearum*, the lesion size and the relative amount of fungal DNA was similar in transgenic vs. wt plants. Consistent with this finding, root lengths and root fresh weights were similar between transgenic and wt plants (**Figure 5**). However, in clear contrast, AtPIIN_08944OE plants showed enhanced susceptibility to *Hpa* compared to wt plants as observed by a higher number of sporangiophores on the true leaves of AtPIIN_08944OE vs. wt plants (**Figure 3D**). These findings support the hypothesis that PIIN_08944 plays a critical role in the Sebacinalean symbiosis by interfering with the plant’s SA-mediated basal resistance response.

## Discussion

In the present work, we have corroborated and extended previous reports showing that mutualistic fungi, like pathogens, use effectors to suppress or circumvent host defense. MiSSP7, an effector of the ectomycorrhizal fungus *Laccaria bicolor*, and SP7, an effector of the arbuscular mycorrhizal fungus *Glomus intraradices*, suppresses host immunity to promote root symbiosis. SP7 targets the pathogenesis-related transcription factor ERF19 in the nucleus, while MiSSP7 targets the repressor proteins JAZ5 and JAZ6 in the nucleus and therefore inhibits the expression of JA-induced genes thereby promotes root colonization by the mycorrhizal fungus (Kloppholz et al., 2011; Plett et al., 2011; Plett et al., 2014).

The genome of *P. indica* contains hundreds of putative effector genes coding for SSPs (Zuccaro et al., 2011). About 123 out of 216 SSPs were responsive to colonization either on *Arabidopsis* or barley (Lahrmann et al., 2013), which also may indicate that colonization on different hosts needs a specialized set of effectors. Interestingly, 21 SSPs were expressed at similar symbiotic stages both in *Arabidopsis* and barley, suggesting that these effector candidates might target similar cellular processes. Our results show that PIIN_08944 is a host unspecific effector which is exploited by *P. indica* to target conserved molecular processes in different plants and therefore act as a general determinant of compatibility during plant root colonization.

The accumulation of *PIIN_08944* transcripts in *in vitro* germinated chlamydospores shows that *PIIN_08944* is induced before direct contact with a host, which also suggests that the induction is not dependent on a plant signal. Moreover, *PIIN_08944* was not differentially expressed in the course of *P. indica*’s colonization of *Arabidopsis* roots, which is consistent with our analysis of public available microarray data (Lahrmann et al., 2013). However, based on the available microarray data, *PIIN_08944* is among the top 20% of highly expressed *P. indica* genes. In support of our finding, the effector AvrM of the rust fungus *Melampsora lini* also was not differentially regulated during colonization of flax, and its expression was independent of a host-derived signal (Catanzariti et al., 2006). One reason for the accumulation of *PIIN_08944* in germinating spores could be that this effector is important to counteract the pre-invasive host defenses and thereby might prepare the host cell for subsequent hyphal colonization. Therefore it is not surprising that the expression of effectors can be induced even before host contact has been established.

The observation that deletion of *PIIN_08944* impaired the ability of *P. indica* to colonize *Arabidopsis* roots suggests that the effector contributes to fungal colonization during the interaction of *P. indica* with its host plant. The loss of virulence was rescued by the *in planta* expression of *PIIN_08944*. This finding also raises the possibility that PIIN_08944 is secreted and operates inside host cells. Furthermore, barley plants expressing *PIIN_08944*, showed enhanced colonization when inoculated with the *P. indica* wt strain, indicating that the protein also contributes to colonization on barley root (**Supplementary Figure S3**).

The ability of PIIN_08944 to suppress flg22-induced defense responses, which was observed by the dampened expression of the marker gene *WRKY22*, suggests that the effector interferes with early PTI signaling. Suppression of the SA marker *CBP60g* is most likely an indirect result of early PTI suppression (Tsuda et al., 2013). This hypothesis is further supported in that AtPIIN_08944OE and HvPIIN_08944OE plants did not resist the necrotrophic fungal pathogens *F. graminearum* and *B. cinerea* as these fungi are highly sensitive to JA-mediated defense responses (Gottwald et al., 2012; Aubert et al., 2015). The phytohormone SA is widely accepted to be involved in resistance against biotrophic pathogens (Glazebrook, 2005). The suppression of the SA marker gene *CBP60g*, might also explain the enhanced susceptibility observed for AtPIIN_08944 plants to *Hpa*. Earlier reports suggested that *P. indica* recruits JA signaling to counter SA associated defense as showed by the up-regulation of *VEGETATIVE STORAGE PROTEIN 2* (*VSP2)*, a marker gene for JA on *P. indica* colonized plants (Jacobs et al., 2011). We speculate that PIIN_08944 is unlikely to affect the JA response as necrotrophic *Bc* infection on *Arabidopsis* AtPIIN_08944OE plants and *Fg* infection on barley HvPIIN_08944OE plants were similar to wt.

Interestingly, flg22- and chitin-mediated ROS production was reduced on HvPIIN_08944OE plants but not in transgenic *Arabidopsis*. This finding might indicate that *P. indica* can exploit PIIN_08944 to suppress early PTI responses triggered by chitin perception during fungal colonization of roots. At present it remains unclear whether there are differences in early PTI signaling events in barley vs. *Arabidopsis*. The differential ROS response mediated by PIIN_08944 in barley vs. *Arabidopsis* may hint to such difference. However, whether PIIN_08944 is able to suppress chitin-induced ROS burst in *Arabidopsis* still needs further investigation.

Taken together, our results show that *P. indica* has evolved effectors such as PIIN_08944 as important determinants that contribute to the establishment and/or maintenance of a mutualistic relationship during interaction with plants. Moreover, the suppressive activity of PIIN_08944 in a dicot (*Arabidopsis*) and a monocot (barley) might give a first hint on our understanding at the molecular level, how *P. indica* can colonize a broad spectrum of different plant species. It is plausible that several other effector candidates may contribute in shaping the colonization process of *P. indica*. Therefore it would be important to investigate the role played by other *P. indica* effector candidates during root colonization.

## Author Contributions

FA and K-HK designed the research. FA and DB performed the experiments. FA, JS, and K-HK analyzed data. FA, JS and K-HK wrote the manuscript.

## Conflict of Interest Statement

The authors declare that the research was conducted in the absence of any commercial or financial relationships that could be construed as a potential conflict of interest.

## Footnotes

1 http://dna-cloning.com/skripte/puc/puc.html

2 http://dna-cloning.com/skripte/binaries/bin.html

3 http://imagej.nih.gov/ij/

4 http://www.ncbi.nlm.nih.gov/Structure/cdd/wrpsb.cgi

5 http://pfam.xfam.org/

6 http://www.ebi.ac.uk/arrayexpress

## Supplementary Material

**FIGURE S1 | Phenotypic analysis of *Piriformospora indica* Pi_Δ08944_ mutants (*Pi*T1 and *Pi*T2) and wt. Fungal growth rates and morphology was similar between Pi_Δ08944_ mutants (*Pi*T1 and *Pi*T2) and the wt fungus**.

**FIGURE S2 | Analysis of HvPIIN_08944OE in barley plants.** Western blot analysis of transgenic barley expressing *GFP: PIIN_08944* fusion protein using anti GFP antibody. No band was observed in wt plants (c), a lower GFP band (∼27 kDa) was observed in the GFP control, and a 39 KDa band corresponding to *GFP: PIIN_08944* was observed in HvPIIN_08944OE plants.

**FIGURE S3 | Enhanced colonization by *P. indica* of barley expressing *PIIN_08944*.** Roots of 3-day-old transgenic barley seedlings or the EV control were inoculated with *P. indica* chlamydospore. Root material was harvested at 3, 7, 14, and 21 dpi; genomic DNA was extracted and the relative amount of fungal biomass was determined by qPCR using plant and fungal specific primers. Data represent the mean with ±SD of two independent experiments. Asterisks indicate significance at ^∗^*P* < 0.05 analyzed using student’s *t-*test.

**FIGURE S4 | Analysis of *P. indica* transgenic mutants and transgenic *Arabidopsis*.** **(A)** Relative expression of *PIIN_08944* in *in vitro* germinated spores of the five Pi_Δ08944_ strains vs. wt by RT-PCR. *PIIN_08944* transcripts accumulated only in wt. The *P. indica ubiquitin UBQ* gene served as reference. **(B)** Determination of T-DNA copy numbers in Pi_Δ08944_ transformants by Southern blot analysis. Genomic DNA from transformants and wt was digested with SacI, separated on 0.9% TAE agarose gel, transferred to a nylon membrane, and hybridized with a ^32^P radio-labeled ^32^P-dCTP 600 bp fragment of hygromycin B (*hph*). Three out of the five transformants have one copy of T-DNA inserted in the genome, while two have two copies. Lanes 1–5: *Pi*T1 *to Pi*T5. **(C)** Expression analysis of *Arabidopsis* expressing *PIIN_08944* (upper panel) and *ubiquitin* UBQ (lower panel) by RT-PCR. #1–#17: independent AtPIIN_08944OE, C: Wt.

**TABLE S1 | List of primers used**.
