# Supplementary figures and images for: The Piriformospora indica effector PIIN_08944 promotes the mutualistic Sebacinalean symbiosis
Source: Front Plant Sci. 2015 Oct 26;6:906. doi: 10.3389/fpls.2015.00906 (PMC4620400; doi:10.3389/fpls.2015.00906)

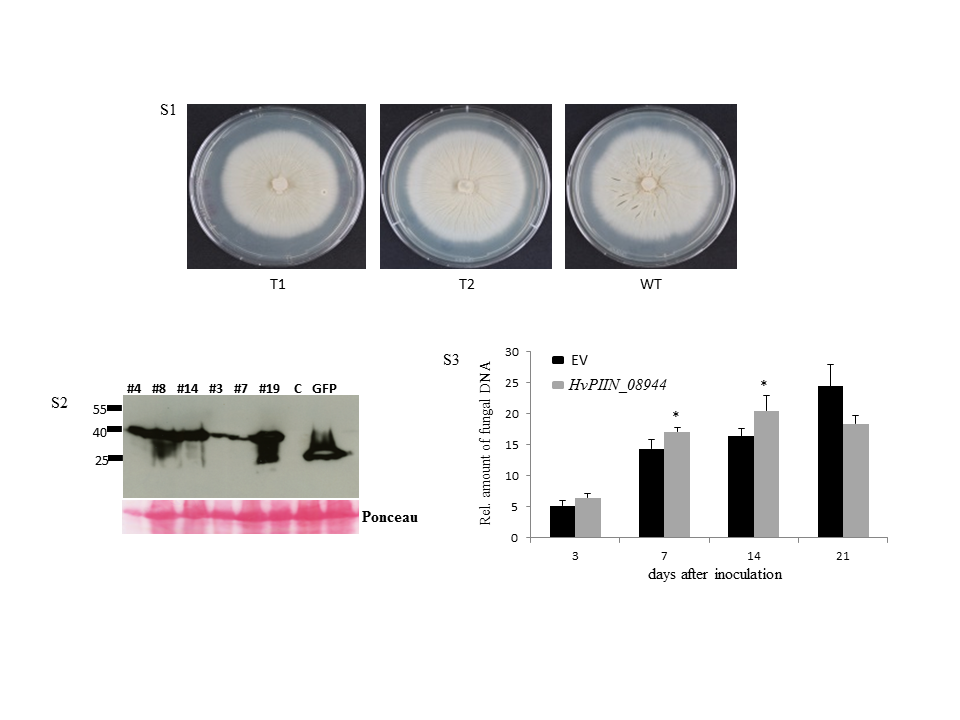

Supplement: Supplementary file 1 [file Image_1.TIF]

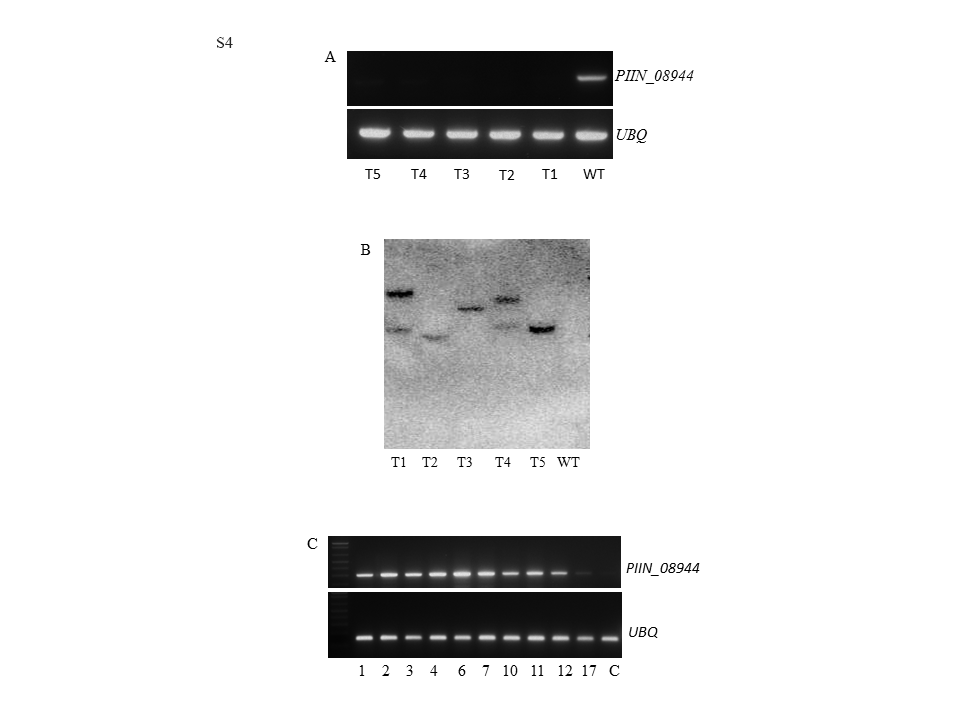

Supplement: Supplementary file 2 [file Image_2.TIF]
