# Supplementary material for: The Piriformospora indica effector PIIN_08944 promotes the mutualistic Sebacinalean symbiosis
Source: Front Plant Sci. 2015 Oct 26;6:906. doi: 10.3389/fpls.2015.00906 (PMC4620400; doi:10.3389/fpls.2015.00906)
Supplement: Supplementary file 3 [file Table_1.DOCX]

**Supplementary Table S1. List of primers used**

| Gene | Acc. Nr | Sequences |
| --- | --- | --- |
| AtUBQ5_FW | AT3G62250 | CCAAGCCGAAGATCAAG |
| AtUBQ5_RV |  | ATGACTCGCCATGAAAGTCC |
| Β-tubulin_F | FGSG_09530 | ATCTCGAGCCCGGTACCATGG |
| Β-tubulin_R |  | CTCGGTGTAATGACCCTTGGCC |
| ITS _F |  | CAACACATGTGCACGTCGAT |
| ITS_R |  | CCAATGTGCATTCAGAACGA |
| WRKY22_F | AT4G01250 | ATCTCCGACGACCACTATTG |
| WRKY22_R |  | TCATCGCTAACCACCGTATC |
| CBP60g_F | AT5G26920 | AAGAAGAATTGTCCGAGAGGAG |
| CBP60g_R |  | GGCGAGTTTATGAAGCACAG |
| Pi_UBI_F | PIIN_01523 | GCAGCTCGAAGATGGTCGCA |
| Pi_UBI_R |  | ACATGCACGCTTGCGGCAGT |
| AtUbQ4_F | [AT5G20620](http://www.arabidopsis.org/servlets/TairObject?type=locus&name=AT5G20620) | GCTTGGAGTCCTGCTTGGACG |
| AtUBQ4_R |  | CGCAGTTAAGAGGACTGTCCGGC |
| KpnI_US_08944_F | PIIN_08944 | TATGGTACCTTCGAGTCCAGGCGACAACG |
| KpnI_US_08944_R |  | GCGGGTACCGTCGATTACGAATTGGTGAAG |
| StuI_DS_08944_F | PIIN_08944 | GTCAGGCCTACCTGTAAAGTAACTCCTTCCCCTC |
| SacI_DS_08944_R |  | GTCGAGCTCGGATAAAACCTTCGAAGGACAGTG |
| Hyg_F | G0FGT3 | TATCGGCACTTTGCATCGGC |
| Hyg_R |  | GATCGGACGATTGCGTCGCA |
| XmaI_08944_F | PIIN_08944 | ATACCCGGGATGTTCTCTTTCCGAAAAGCCGCA |
| NotI_08944_R |  | TTTGCGGCCGCTGATTCGTGAGTTTTACGCTTTCCG |
